# Evidence base for psychological treatment of personality disorder – a narrative review

**DOI:** 10.1192/bjo.2021.739

**Published:** 2021-06-18

**Authors:** Caoimhe Ni Shuilleabhain

**Affiliations:** Camden and Islington NHS Trust

## Abstract

**Aims:**

This review critically appraises the up-to-date evidence base for psychological treatment of PD.

**Background:**

The prevalence rate of any personality disorder (PD) in the general population has been estimated to be as high as 12% rising to over 70% in prison settings. PD is known to carry significant psychosocial and health burdens with increased mortality, increased suicide, increased substance misuse, increased crime, reduced capacity to work, poorer outcomes for comorbid mental disorders, dysfunctional engagement with services, and high economic costs through a high utilisation of healthcare systems. In the 1990s several manualised treatment strategies emerged, specifically for borderline PD. These include dialectical behaviour therapy, cognitive therapy, cognitive analytic therapy, mentalization-based therapy, transference-focused psychotherapy, and schema-focussed therapy.

**Method:**

Using relevant search criteria, literature was identified through a search of the following databases: PubMed, EMBASE, and PsycINFO. Data were appraised and synthesised to provide a comprehensive overview of the current evidence base for psychological treatment of PD.

**Result:**

The DSM-V defined Cluster B borderline PD has received the majority of attention. Increasing attention has been paid in recent years to the Cluster B antisocial PD. Cluster A (Paranoid, Schizoid, Schizotypal) and Cluster C PDs (Avoidant, Dependent, Obsessive Compulsive) have received relatively little attention with few studies to draw upon regarding the effectiveness of therapy. The remaining Cluster B personality disorders (Narcissistic and Histrionic) have been criticised as having poor construct validity, with a lack of rigorously designed treatment trials.

A number of treatment protocols have gained empirical support. However, of those that have empirical support, there appears to be little demonstrable evidence to suggest superiority of any one of the evidence-based interventions over another. While specialised therapies are more efficacious than “treatment as usual” or treatment delivered by expert clinicians, when specialised therapies are compared with well-specified manualised general psychiatric care tailored to personality disorder, the results are different, with little consistent evidence demonstrating the superiority of specialised therapies.

**Conclusion:**

Current evidence suggests that individual therapies do not differ substantially from each other or from structured clinical care that relies on generic change factors. This is in keeping with established psychotherapy outcome literature. Current evidence would indicate that common features across the proven treatment strategies should be emphasised and implemented well. There may be justification for added interventions from specific treatment modalities targeted to specific patient problems.

